# A QuEChERS-Based Liquid Chromatography-Tandem Mass Spectrometry Method for the Simultaneous Determination of Nine Zearalenone-Like Mycotoxins in Pigs

**DOI:** 10.3390/toxins10030129

**Published:** 2018-03-20

**Authors:** Zheng Yan, Lan Wang, Jun Wang, Yanglan Tan, Dianzhen Yu, Xiaojiao Chang, Yingying Fan, Duoyong Zhao, Cheng Wang, Marthe De Boevre, Sarah De Saeger, Changpo Sun, Aibo Wu

**Affiliations:** 1 SIBS-UGENT-SJTU Joint Laboratory of Mycotoxin Research, Key Laboratory of Food Safety Research, Shanghai Institutes for Biological Sciences, University of Chinese Academy of Sciences, Chinese Academy of Sciences, Shanghai 200000, China; zyan@sibs.ac.cn (Z.Y.); wanglan@sibs.ac.cn (L.W.); yanglantan@gmail.com (Y.T.); dzyu@sibs.ac.cn (D.Y.); 2 Academy of State Administration of Grain P.R.C, No. 11 Baiwanzhuang Avenue, Xicheng District, Beijing 100037, China; wj@chinagrain.org (J.W.); cxj@chinagrain.org (X.C.); 3 Key Laboratory of Agro-Products Quality and Safety of Xinjiang/Laboratory of Quality and Safety Risk Assessment for Agro-Products (Urumqi), Ministry of Agriculture/Institute of Quality Standards & Testing Technology for Agro-Products, Xinjiang Academy of Agricultural Sciences, Urumqi 830091, China; fyyxaas@sina.com (Y.F.); luckydyz@163.com (D.Z.); xjzbs2018@sina.com (C.W.); 4 Laboratory of Food Analysis, Department of Bioanalysis, Faculty of Pharmaceutical Sciences, Ghent University, 9000 Ghent, Belgium; Marthe.DeBoevre@UGent.be (M.D.B.); Sarah.DeSaeger@UGent.be (S.D.S.)

**Keywords:** zearalenone, LC-MS/MS, QuEChERS, pigs

## Abstract

The determination of zearalenone (ZEN) and its derivatives as biomarkers in animal tissues or organs plays an important role in mycotoxin monitoring and can promote effective exposure assessment. A liquid chromatography-tandem mass spectrometry (LC-MS/MS) method for the simultaneous quantification of nine ZEN-like mycotoxins, including three glucuronides in different pig tissues (heart, liver, spleen and muscle) was developed and validated in this study. Tissue samples were extracted using a quick, easy, cheap, effective, rugged, and safe (QuEChERS) extraction and clean-up procedure, and analyzed by LC-MS/MS in multiple reaction monitoring (MRM) mode. Dynamic linear ranges for each target analyte were determined with *R*^2^ between 0.916 and 0.999. The LODs of the six ZENs were achieved in the range of 0.5–1 ng/g and the LOQs varied from 1 ng/g to 2 ng/g. The satisfying intra-day and inter-day reproducibility (both RSD_r_ and RSD_R_ < 20%) indicated a good stability of this method. The recoveries of the nine target analytes were in the range of 70–110%. The validation results showed that this LC-MS/MS method coupled with QuEChERS sample pretreatment is effective and suitable for the simultaneous quantitation of ZEN metabolites in pigs. It has been applied to analysis of the pig tissues in this research and can be also adapted for samples in the mycotoxin research field.

## 1. Introduction

Mycotoxins are toxic secondary metabolites produced by various molds or fungi which are commonly found in feed and foodstuff. Crops, such as corn, wheat and corn are easily contaminated by mold during growth, harvest and storage [1]. Fungal mycotoxins are produced in the growth process and exert great toxicity to human and animals. It is considered a public concern because they have the potential to affect the animals either individually or additively in the presence of more than one mycotoxin. Subsequently, mycotoxins may cause harm to various organs such as spleen, liver, heart and immune system, eventually resulting in production reduce of the animals and causing economic losses [2].

**Figure 1:**
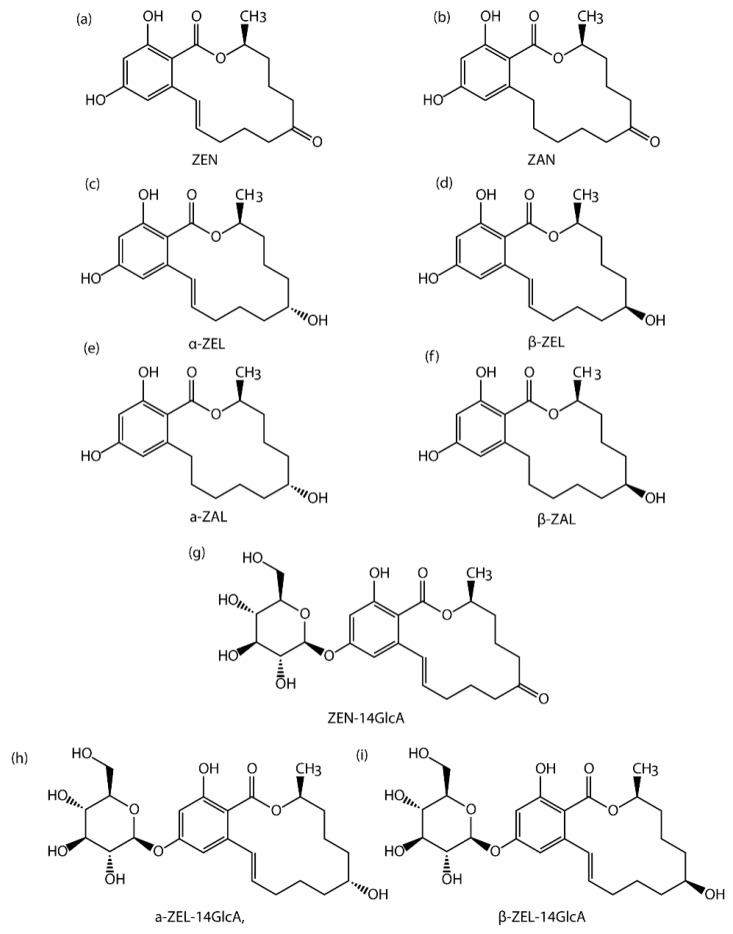
Chemical structure of 9 mycotoxins. (**a**) ZEN (**b**) ZAN (**c**) α-ZEL (**d**) β-ZEL (**e**) α-ZAL (**f**) β-ZAL (**g**) ZEN-14GlcA (**h**) α-ZEL-14GlcA (**i**) β-ZEL-14GlcA.

Among all mycotoxins, Zearalenone (ZEN, Figure 1), which is a non-steroidal estrogenic mycotoxin produced by *F. culmorum* and *F. graminearum*, has several adverse effects [3,4,5,6,7]. Occurrence, toxicity, and metabolism data of ZEN was summarized by the European Food Safety Authority and in several reviews [8,9,10,11]. Subsequently, ZEN was associated with hyperestrogenism and several physiological alterations of the reproductive tract such as infertility and low hormone levels in several laboratory animals (mice, rat, pig) [12]. ZEN has oestrogenic effects on livestock by binding to the oestrogen receptors ER-α and ER-β, which can cause infertility and reduce weight or embryonic death [13]. Although their modes of action are different, pigs are more sensitive to this toxin [14].The target organ of ZEN is mainly the reproductive system of female animals. While at the same time, it also has certain influence on male animals. In the condition of acute poisoning, it will have some toxic effects on the nervous system, heart, kidney, liver and lung [15].

Additionally, animal data suggest that ZEN biotransformation occurs in the liver & intestinal tissues, and lead to generation of five metabolites, including, zearalenone (ZAN, Figure 1), β-zearalanol (β-ZAL, Figure 1), α-zearalanol (α-ZAL, Figure 1), β-zearalenol (β-ZEL, Figure 1), α-zearalenol (α-ZEL, Figure 1) [13,16,17]. On the other hand, several studies showed that these metabolites are glucuronidated in the organs, preferably at the sterically unhindered 14-hydroxyl-position, and generate conjugated forms [18,19] such as β-zearalenol-glucuronide (β-ZEL-14G, Figure 1), α-zearalenol-glucuronide (a-ZEL-14G, Figure 1), zearalenone-14-glucuronide (ZEN-14GlcA, Figure 1) in animals. In the recent study, ZEN-14-GlcA was the predominant glucuronide, moreover the glucuronidation rate of ZEN in pigs is likely to be faster than in other species [18]. Several studies have revealed that ZEN and its main metabolites have anabolic activities in farm animals, especially in pigs [20,21,22]. Typical symptoms described in swine and piglets are hyperemia, swelling and reddening of the vulva in gilts, enlargement of the mammary glands and edema of the vagina [23]. A survey in 2001 showed that clinical symptoms of ZEN could be severed by the consequence of additive effects exerted by both ZEN and metabolic compounds [24]. On the other hand, several evidence demonstrated that ZEN as well as its metabolites are able to cause serious health effects in pigs, which not only lead to fertility disorders but also disturb the ovulation cycle and reduce litter size in swine [25]. Thus, serious impact on profit may be registered by swine producers as a result of reproductive failure related to the presence of ZEN and its metabolites in feedstuff [26]. However, in contrast to ZEN, no maximum or regulatory limits have been set for its modified forms as the consequence of the lack of occurrence and toxicity data. To date, there is no valid method for determining these modified forms, especially in animal-derived food. Therefore, the European Food Safety Authority (EFSA) recently addressed the need to set a properly validated and sensitive analytical method for detecting the modified forms of mycotoxins in these matrices [26].

Since contamination by fungal toxin frequently occur in food, regulatory limits for ZEN have been established in many countries. Maximum permitted levels for ZEN in different foodstuffs, introduced by EU are 350 μg/kg in unprocessed corn. The EFSA Panel on Contaminants in the Food Chain (CONTAM) set for a no-observed-effect-level (NOEL) of 10 μg/kg bw/day for pigs and published a tolerable daily intake (TDI) for humans of 0.25 µg/kg bw/day.

In recent years, the QuEChERS (quick, easy, cheap, effective, rugged, and safe) method is a widely used sample preparation methodology. The method involves extraction with acetonitrile and partitioning cleanup after the addition of a salt mixture (MgSO_4_ and NaCl). Clean-up is also performed by solid-phase extraction through the addition of appropriate sorbents. As a result, this method overcome obstacles such as high-cost, requiring skilled operation technique, low detection levels as well as time-consuming. Therefore, it has been applied in analytical fields from drugs to mycotoxins [27,28,29]. 

Based on its toxic action as well as the economic problem caused by contaminated feedstuff, both ZEN and its reduced metabolites should be monitored. In order to ensure people’s safety, it is necessary to establish effective methods to detect toxins. During the past few years, a various number of analytical methods such as enzyme-linked immunosorbent assay (ELISA), thin layer chromatography (TLC), high-performance liquid chromatography (HPLC) and gas chromatography-tandem mass spectrometry (GC-MS/MS) have been developed for the identification and quantification of ZEN and other mycotoxins [30,31,32,33,34,35,36]. The HPLC-application is limited due to unsatisfactory accuracy of quantification and poor separation, therefore LC/MS replaced HPLC and became the most commonly used method for ZEN analysis [37,38,39]. For example, Recently, Breidbach (2017) developed a greener, more quick approach for the determination of multiple mycotoxins including ZEN using LC-MS/MS [40].

Pigs, especially weaning piglets, are a good model to investigate the toxic effects of ZEN and its metabolites because of (1) the high percentage of cereals in their diet and (2) they are highly sensitive to these compounds [26,41]. Also, ZEN-contaminated diets induced significant changes on the global transcriptome in pig spleen, liver and other organs. Several evidences have demonstrated that the assessment of animal exposure to ZEN and its modified compounds might not only analyze toxin residues in feedstuff, but also analyze the biological samples such as urine, blood, bile, spleen and liver because in most cases, the feedstuffs cannot clearly be related to the disease risk of the animals [8,38,42]. Therefore, determination of ZEN and its derivates in different tissues can serve as biomarkers and could promote effective exposure assessment which is significant for the establishment of regulatory limits. Biomarker research in ZEN and its metabolites started from the 1990s. However, methods for the simultaneous determination of ZEN and its main modified forms in biological samples are scarce with the exception of blood for their availability and convenience [30,43]. No experiments are available describing the bioavailability from contaminated biological samples which presumably contain modified ZEN, especially the glycosidic bound ZEN forms (ZEN-14GlcA). The call for the diagnosis of ZEN-like mycotoxins exposure of farm animals especially pigs becomes louder.

The prerequisite to investigate the metabolism and kinetics of ZEN family mycotoxins is the development of analytical methods for the determination of the toxins in biological matrices such as liver, spleen, urine and muscle. In this paper, our aim is to develop and validate an LC-MS/MS-based QuEChERS method for the simultaneous quantification of ZEN family mycotoxins including ZEN and most relevant key metabolites in different tissues or organs (heart, liver, spleen and muscle) from pigs. It is the first time that a method is developed for the determination of β-ZEL-14G, α-ZEL-14G, ZEN-14GlcA in organs from pigs. This method has been successfully applied in biological samples contaminated by ZEN family. The determination of the nine mycotoxins in four different tissues were revealed through this method. Thus, for the first time, a quick, easy, cheap and effective LC-MS/MS method for the simultaneous determination of both ZEN and its key metabolites in four different type of tissue from pigs was successfully developed.

## 2. Results and Discussion

### 2.1. Selectivity

**Figure 2:**
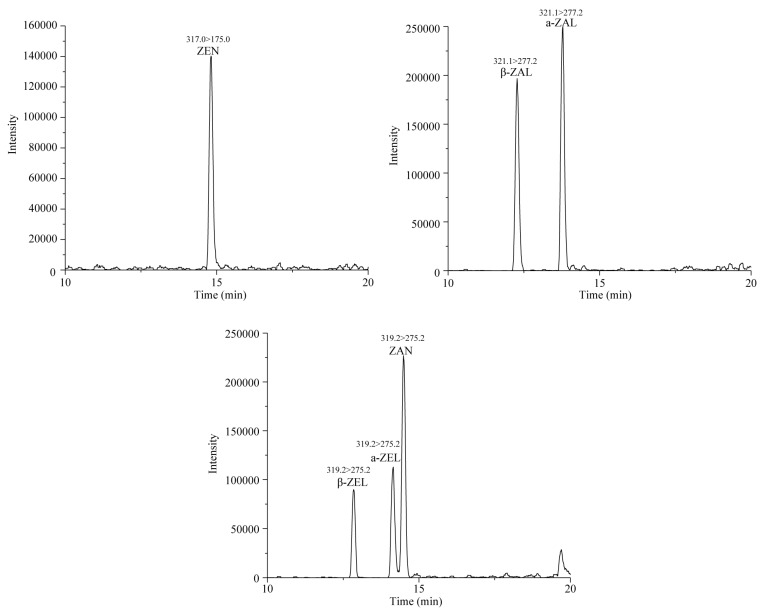
LC-MS/MS chromatograms of ZEN, ZAN, α-ZEL, β-ZEL, α-ZAL and β-ZAL at 20 ng/mL.

**Figure 3:**
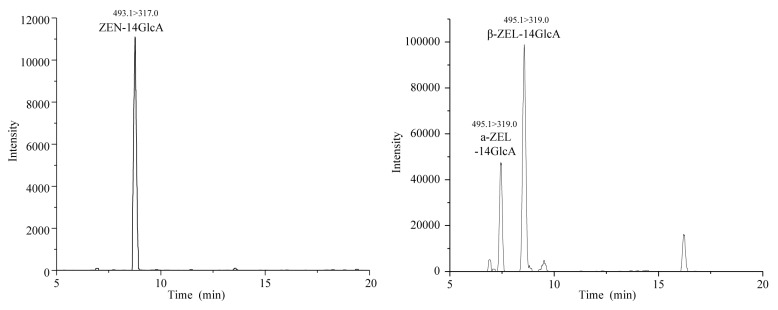
LC-MS/MS chromatograms of ZEN-14GlcA, α-ZEL-14GlcA and β-ZEL-14GlcA at 20 ng/mL.

The selectivity of the method was investigated to evaluate matrix interferences at the retention time in blank samples. The retention time of six target mycotoxins including ZEN, ZAN, β-ZAL, α-ZAL, β-ZEL, α-ZEL are pointed out by chromatograms of a standard mixture in Figure 2, while the ZEN-14GlcA, β-ZEL14GlcA, α-ZEL14GlcA are showed in Figure 3. The retention time in the chromatograms of the blank samples show high levels of reproducibility without interfering peaks, indicating the good selectivity of the method.

### 2.2. Matrix Effect

Due to the complexity of biological samples, the matrix often has significant interferences on the electrospray ionization (ESI) process, which affects the accuracy of mass spectrometric analyses. These effects and disturbances are termed as matrix effects. Therefore, it is necessary to evaluate the matrix effects of different tissues in mycotoxin detection; signal suppression and enhancement (SSE) was calculated, as follows

SSE = A/C × 100(1)

With A = the slope of the calibration curve in matrix, C = the slope of the calibration curve in neat solvent. Generally, SSE is considered tolerable when it varies from 80 to 120%. As shown in Table 1, SSE in liver and muscle was between 80% and 120% indicating that there is no obvious matrix effect. However, significant signal suppression was observed for heat and spleen samples, giving SSE below the lower limit criterion (80%). No signal enhancement was shown in all tissues samples. In order to minimize the matrix effect and to ensure the precision of results, it is essential to establish the matrix-matched calibration curves.

### 2.3. Linearity and Sensitivity of Method

The linear regression equation is analyzed by using the peak areas obtained from each analyte and the sequences of concentration. The result showed that there is a nice linear relationship between 0.916 and 0.999 within their linear ranges. An overview of linearity, correlation coefficients of six mycotoxins in different tissues from pigs are shown in Table 1.

The sensitivity of this method was assessed by measuring limits of detection (LOD) and limits of quantification (LOQ) in this study. The difference between the two is that the minimum concentration specified in the LOQs should meet the requirements of precision. As shown in Table 2, the obtained LODs and LOQs of the ZEN mycotoxins were in the range of 0.5 to 1 ng/g and 1 to 2 ng/g in the heart, liver, spleen and muscle of pigs. The results in our study were similar to the results that the LOD of ZEN and its metabolites varied between 0.1 ng/g and 3 ng/g for tissue samples HPLC-MS/MS [44].

### 2.4. Intra-Day and Inter-Day Precision

The intra- and inter-day precision (%RSD) were evaluated using standard addition. Here, we use the RSD_r_ to represent intra-day precision and RSD_R_ to represent inter-day precision. In this experiment, low (5 ng/mL), intermediate (10 ng/mL) and high (20 ng/mL) concentration levels were selected and analyzed. The precision data showed that the RSD_r_ values in different tissues were less than 15%. The RSD_R_ values in different tissues were higher than RSD_r_ but were still no more than 20%, which demonstrated the stability of this method [45]. These results are summarized in Table 3.

### 2.5. Extraction Recovery

For the evaluation of the recovery, standard addition method was employed in this study. Non-contaminated matrix samples (heart, liver, spleen and muscle) were spiked with nine mycotoxins (ZAN, ZEN, β-ZAL, α-ZAL, β-ZEL, α-ZEL, ZEN-14GlcA, β-ZEL-14GlcA, α-ZEL-14GlcA) at low (5 ng/mL), intermediate (10 ng/mL) and high (20 ng/mL) concentration levels, and pretreated using the method present in Section 4.3. After analysis, the method extraction recovery (RE) was calculated as:

RE = B/D × 100(2)

**Figure 4:**
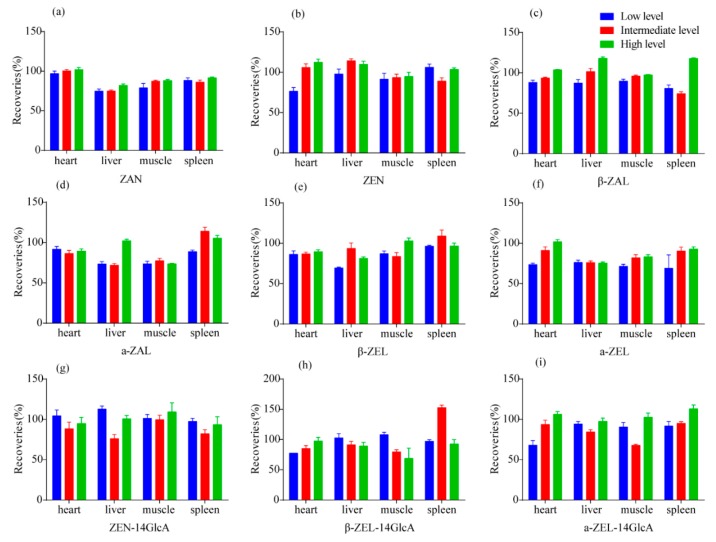
Comparison of the recoveries of nine mycotoxins in different tissues (heart, liver, muscle and spleen). The high level was 20 ng/mL; Intermediate level was 10 ng/mL; Low level was 5 ng/mL. (**a**) ZAN (**b**) ZEN (**c**) β-ZAL (**d**) α-ZAL (**e**) β-ZEL (**f**) α-ZEL (**g**) ZEN-14GlcA (**h**) β-ZEL-14GlcA (**i**) α-ZEL-14GlcA.

B = the slope of sample spiked after extraction, D = the slope of sample spiked before extraction. The extration recoveries of all the spiked tissues of the nine target toxins were between 70% and 110%, suggesting that the pretreatment method met the requirement of mycotoxin determination in the different pig tissues [45]. These results are pointed out in Figure 4.

### 2.6. Analysis of Real Samples

The established QuEChERS pretreatment and LC-MS/MS method was applied and validated for the analysis of 16 pig tissue samples, including 4 live samples, 4 heart samples, 4 spleen samples and 4 muscle samples muscle (n_total_ = 16). The results are given in Table 4, showing that the method had been successfully applied. In the heart sample, ZEN, ZAN and α-ZAL were not detected while the level of two modified mycotoxins were significantly higher. Similarly, in the spleen sample, it was observed that there were abundant more modified mycotoxins including α-ZEL-14GlcA, β-ZEL-14GlcA and ZEN-14GlcA.However, ZEN, α-ZAL and β-ZAL were barely detected. These results might provide a potential to find a reliable biomarker of exposure. In addition, in the liver sample, only ZAN, ZEN-14GlcA and β-ZEL-14GlcA were detected. While ZEN, β-ZEL, α-ZEL, β-ZAL, α-ZAL and α-ZEL-14GlcA were not detected. There was only a small amount of ZAN in the muscle samples. The result of all mycotoxins proved the validity of our method in this study.

## 3. Conclusions

At present, there are many researches on ZEN in food and feed crops, plants and herbs. However, till now only few studies have been executed on animals. The challenge for exposure assessment is that not only the parent toxin has to be detected, but also the metabolites should be considered, especially ZEN-14GlcA, β-ZEL-14GlcA and α-ZEL-14GlcA. Quantitation of ZEN-like mycotoxins including its key metabolites in biological samples does not enable evaluation of ZEN exposure in pig, but also allows the risk assessment for human health. In this study, we have successfully validated and applied an LC-MS/MS based method coupled with a QuEChERS pretreatment for the simultaneous quantification of ZEN-like mycotoxins including ZEN and its most relevant key metabolites in tissues from different organs (heart, liver, spleen and muscle) in pigs. We detected the modified forms of ZEN including ZEN-14GlcA, β-ZEL-14GlcA, α-ZEL-14GlcA in different animal tissues for the first time. The results showed that there is a linear relationship between 0.916 and 0.999 within their linear ranges. The recoveries of all the spiked tissues of the nine target toxins were between 70% and 110%, suggesting that the pretreatment method met the requirement of mycotoxin determination in the different pig tissues. This method has been successfully applied in real samples contaminated by ZEN-like mycotoxins. In our study, ZEN family especially modified mycotoxins were clearly detected in the pig tissues except muscle sample. The results showed that ZEN was absent or in low concentration, and ZAN was detected in all tissues except heart. The results noted in pig’s tissues exposed to low doses of ZEN were consistent with research findings in female wild boars [11]. The amounts of ZEN-14GlcA and β-ZEL-14GlcA were high in heart, liver and spleen. Since ZEN glucuronides were not found in crops, so the results indicate that ZEN glucuronides are pig metabolites transformed from ZEN. On the one hand, localization of each mycotoxin in different studied tissues may provide the information to study metabolism and discover biomarkers of ZEN mycotoxins in pig. On the other hand, this quick, easy and effective assay will contribute to ZEN-biomarker determination and will promote its exposure assessment.

## 4. Materials and Methods

### 4.1. Chemical and Apparatus

The solid standards of ZEN, ZAN, β-ZAL, α-ZAL, β-ZEL, α-ZEL and C^13^-ZEN were obtained from Sigma (St. Louis, MO, USA) and stored at −20 °C before use. The standards of ZEN-14GlcA, β-ZEL-14GlcA, α-ZEL-14GlcA were provided by the lab of Dr. Bart Huyberchts at CODA-CERVA. All organic solvents, salts and acid were analytical or HPLC grade. Acetonitrile, methanol, hexane, and isopropanol were purchased from Honeywell. Ammonium acetate and formic acid were purchased from Sigma. Milli-Q-quality water (Millipore, Billerica, MA, USA) was used throughout the experiments. C18-columns were obtained from Agela Technologies (Tianjin, China). 

### 4.2. Preparation of Standard Solutions

The standards of ZAN, β-ZAL, α-ZAL were accurately weighed, and dissolved in pure acetonitrile to stock solutions at 10 μg/mL. The standards of ZEN, β-ZEL, α-ZEL were prepared in acetonitrile at a concentration of 1, 2, 5, 10, 20, 50, 100 ng/mL. The solution of C13-ZEN (100 ng/mL) was prepared. All standard solutions were freshly prepared.

### 4.3. Sample Pretreatment

The heart (*n* = 4), liver (*n* = 4), spleen (*n* = 4), muscle (*n* = 4) samples from pigs were provided by the Academy of State Administration of Grain P.R.C. The blank samples (heart, spleen, liver, muscle) were obtained from local supermarket in Shanghai and verified with ZENs not detectable. All samples were cut into the slice around 10 g, and then stored at −80 °C before pretreatment. Sample preparation protocols based on the QuEChERS method were used in the study. Each tissue was homogenized for 120 s on an automatic fast sample grinding instrument. The homogenized sample of 1 g was accurately weighed, mixed with 5 mL water and 5 mL of acetonitrile (formic acid 0.1%) and shaken for 2 min. It was then placed into an ultrasonic extractor for 30 min. Thereafter 2 g of MgSO_4_ and 0.5 g of NaCl were subsequently added, followed by shaking for 2 min. The samples were centrifuged for 5 min at 4000 rpm at room temperature. The supernatant of 1 mL was mixed with 50 mg of C18 and 100 mg of MgSO_4_, shaken for 2 min, and centrifuged at 4000 rpm for 5 min. Then, the supernatant was collected and dried under a gentle stream of nitrogen gas at 50 °C. The residue was dissolved in 400 μL of acetonitrile/water (50:50, *v*/*v*), and mixed by 400 μL of hexane. After centrifugation at 5000 rpm for 5 min, the aqueous phase was collected, and passed through the 0.22 μm nylon filter for analysis.

### 4.4. LC-MS/MS Analysis

The LC-MS/MS system (TSQ Vantage) was utilized for the simultaneous determination of mycotoxins. The chromatographic column used in this method was an Agilent Extend-C_18_ (100 mm × 4.6 mm, 3.5 μm) column with a flow rate of 0.35 mL/min at 30 °C. The sample injection volume was 10 μL. The mobile phase was composed of phase A (5 mM ammonium acetate) and phase B (methanol). The elution program was set as follows: 5%B (initial), 5–15%B (0–2 min), 15–50%B (2–5 min), 50–100%B (5–20 min), 100%B (20–22 min), 100–5%B (22–22.1 min), and held a further 4 min for re-equilibration. The flow rate was 350 μL/min and the total run time was 25 min. Mass spectrometry analysis was carried out in both positive and negative ionization mode using multiple reaction monitoring (MRM). The optimized conditions were used for analysis: the vaporizer temperature 300 °C; the ion transfer tube temperature 350 °C spray voltage of negative ESI 3 KV; sheath gas pressure 30 psi; aux flow 15 arb; collision gas (argon) pressure 1.5 mTorr. Table 5 summarizes the optimized MS/MS parameters of the nine mycotoxins in MRM mode.

### 4.5. Method Validation

Matrix effect, linearity, limit of detection (LOD), limit of quantification (LOQ), intra- and inter-day precision and extraction recovery were evaluated blank tissues (heart, spleen, liver as well as muscle) were used for all spiking experiments to validate the method in this study.

#### 4.5.1. Matrix Effect

The matrix effect was measured by calculating the ratio of the slope of the spiked extract (A) and pure standard (C). The analysis value of the standard solution was considered as 100%. The signal suppression and enhancement were used to estimate the matrix effect.

#### 4.5.2. Calibration and Linearity

The linearity was evaluated using matrix-matched calibration curves by spiking blank tissues at 3 concentrations. Calibration curves were constructed by concentration sequences of each analyte. The concentration ranges for all of the analytes were as follows: ZEN, ZAN, β-ZAL, α-ZAL, β-ZEL, α-ZEL at 1–1000 ng/g; ZEN-14GlcA, β-ZEL-14GlcA, α-ZEL-14GlcA at 1–100 ng/g. The coefficient of determination (*R*^2^) was calculated by means of the least-square approach.

#### 4.5.3. Limits of Detection (LOD) and Limits of Quantification (LOQ)

The LOD refers to the lowest concentration component or the minimum quantity that can be detected through the analysis method under defined experimental conditions. The LOQ refers to the lowest concentration component or the minimum quantity of samples to be measured that can be detected through the analysis method. In our study, by determining with serial dilution, the LOD is the minimum concentration giving a detectable signal [qualifying ion S\N~3], whereas the LOQ is the minimum concentration giving a solid peak for quantitation [quantifying ion S\N~10].

#### 4.5.4. Intra-Day and Inter-Day Precision

With respect to intra- and inter-day precision of the method, Low, intermediate and high concentration levels of mycotoxins (5, 10, 20 ng/mL for ZEN, ZAN, β-ZAL, α-ZAL, β-ZEL, α-ZEL, β-ZEL-14GlcA, α-ZEL-14GlcA, ZEN-14GlcA) were spiked into the blank samples (*n* = 3), followed by pretreatment as described in Section 4.3. The intra-day and inter-day reproducibility was calculated as relative standard deviation (RSD_r_ and RSD_R_) by analyzing the samples in triplet every day and in continuous three days.

#### 4.5.5. Recovery

The recovery was assessed by comparing the peak area of blank samples where nine mycotoxins had been added before and after the extraction process at three different concentrations (5 ng/mL, 10 ng/mL, 20 ng/mL). The ratio can be subject to evaluate the recovery of the method.

## Figures and Tables

**Table 1:** Linearity, correlation coefficients and SSE of six mycotoxins in different matrices from pigs.

Matrices	Mycotoxins	Linear Range (ng/mL)	Slope	Correlation Coefficient (*R*^2^)	SSE (%)
Heart	ZEN	2–1000	23,442	0.938	59
	ZAN	2–1000	26,990	0.916	71
	β-ZAL	2–1000	40,729	0.998	88
	α-ZAL	2–1000	46,669	0.997	77
	β-ZEL	2–1000	13,126	0.952	75
	α-ZEL	2–1000	18,008	0.947	56
Liver	ZEN	2–1000	39,596	0.964	100
	ZAN	2–1000	44,744	0.999	118
	β-ZAL	2–1000	54,313	0.998	111
	α-ZAL	2–1000	66,595	0.995	110
	β-ZEL	2–1000	14,801	0.999	85
	α-ZEL	2–1000	25,781	0.999	80
Spleen	ZEN	2–1000	17,170	0.988	43
	ZAN	2–1000	23,789	0.983	63
	β-ZAL	2–1000	32,231	0.979	66
	α-ZAL	2–1000	39,013	0.981	65
	β-ZEL	2–1000	12,221	0.999	70
	α-ZEL	2–1000	18,340	0.973	57
Muscle	ZEN	2–1000	34,379	0.964	88
	ZAN	2–1000	40,070	0.956	106
	β-ZAL	2–1000	49,359	0.999	101
	α-ZAL	2–1000	65,613	0.968	108
	β-ZEL	2–1000	18,425	0.999	106
	α-ZEL	2–1000	28,078	0.972	88

**Table 2:** Limits of detection and quantification of six mycotoxins in four tissues from pigs. (Unit: ng/g).

Toxins	Heart		Liver		Spleen		Muscle	
	LOQ	LOD	LOQ	LOD	LOQ	LOD	LOQ	LOD
ZEN	2	1	2	1	2	1	2	1
ZAN	1	0.5	1	0.5	1	0.5	1	0.5
β-ZAL	1	0.5	1	0.5	1	0.5	1	0.5
a-ZAL	1	0.5	1	0.5	1	0.5	1	0.5
β-ZEL	1	0.5	1	0.5	1	0.5	1	0.5
a-ZEL	1	0.5	1	0.5	1	0.5	1	0.5

**Table 3:** Intra-day and inter-day precision in different tissues (*n* = 3).

Toxins	Heart		Liver		Spleen		Muscle	
	RSD_r_	RSD_R_	RSD_r_	RSD_R_	RSD_r_	RSD_R_	RSD_r_	RSD_R_
ZEN	10.4	11.9	11.3	16.4	11.1	15.1	10.8	11.7
ZAN	7.5	3.8	5.8	10.2	8.3	8.5	0.9	5.5
β-ZAL	9.1	9.4	5.6	9.1	6.2	14.3	6.0	6.5
a-ZAL	8.3	10.5	10.4	15.3	6.8	17.2	8.4	11.4
β-ZEL	5.8	8.2	8.3	18.4	4.6	7.2	8.2	8.6
a-ZEL	9.0	11.1	11.2	18.2	7.5	10.4	11.2	12.2
ZEN-14GlcA	4.6	6.2	8.1	11.9	11.5	14.7	7.1	9.0
β-ZEL-14GlcA	11.1	14.3	11.9	12.2	11.4	14.2	14.1	15.3
α-ZEL-14GlcA	6.9	9.0	6.4	9.3	8.2	15.1	8.6	9.2

**Table 4:** Mycotoxins detected in the pig samples. ND = not detected (<LOD). (Unit: ng/g).

**Mycotoxins**	**Sample1 ^A^**	**Sample2 ^A^**	**Sample3 ^A^**	**Sample4 ^A^**
ZEN	ND	ND	ND	ND
β-ZEL	ND	9.49	8.43	1.88
α-ZEL	2.27	1.68	2.12	<LOQ
ZAN	ND	ND	ND	6.02
β-ZAL	0.87	ND	ND	ND
α-ZAL	ND	ND	ND	ND
ZEN-14GlcA	<LOQ	9.07	22.91	18.32
β-ZEL-14GlcA	1.21	2.36	5.06	5.31
α-ZEL-14GlcA	4.35	ND	ND	ND
^A^ ZEN-like mycotoxins detected in heart sample from pigs.				
**Mycotoxins**	**Sample1 ^B^**	**Sample2 ^B^**	**Sample3 ^B^**	**Sample4 ^B^**
ZEN	<LOQ	ND	ND	ND
β-ZEL	2.40	ND	11.04	12.81
α-ZEL	2.09	6.73	ND	ND
ZAN	11.54	11.77	4.58	<LOQ
β-ZAL	<LOQ	ND	ND	ND
α-ZAL	ND	ND	ND	ND
ZEN-14GlcA	47.81	18.68	6.46	14.99
β-ZEL-14GlcA	40.92	28.13	1.49	1.30
α-ZEL-14GlcA	17.80	5.34	ND	ND
^B^ ZEN-like mycotoxins detected in spleen sample from pigs.				
**Mycotoxins**	**Sample1 ^C^**	**Sample2 ^C^**	**Sample3 ^C^**	**Sample4 ^C^**
ZEN	ND	ND	ND	ND
β-ZEL	ND	ND	ND	ND
α-ZEL	1.55	ND	ND	ND
ZAN	4.26	4.94	4.66	4.12
β-ZAL	ND	ND	ND	ND
α-ZAL	ND	ND	ND	ND
ZEN-14GlcA	1.60	6.09	17.77	11.37
β-ZEL-14GlcA	<LOQ	1.12	4.20	3.27
α-ZEL-14GlcA	ND	ND	ND	ND
^C^ ZEN-like mycotoxins detected in liver sample from pigs.				
**Mycotoxins**	**Sample1 ^D^**	**Sample2 ^D^**	**Sample3 ^D^**	**Sample4 ^D^**
ZEN	ND	ND	ND	ND
β-ZEL	ND	ND	ND	ND
α-ZEL	ND	ND	ND	<LOQ
ZAN	3.85	5.56	4.33	5.12
β-ZAL	ND	ND	ND	ND
α-ZAL	ND	ND	ND	ND
ZEN-14GlcA	<LOQ	ND	ND	ND
β-ZEL-14GlcA	ND	ND	ND	ND
α-ZEL-14GlcA	ND	ND	ND	ND
^D^ ZEN-like mycotoxins detected in muscle sample from pigs.				

**Table 5:** MS/MS parameters of nine mycotoxins in MRM mode.

Toxins	Precursor Ion (m/z)	Product Ions (m/z)	Collision Energy (eV)	Retention Time (min)	Ratio ^2^ (%)
ZEN	317.0	175.0 ^1^	25.0	15.4	70.5
		131.0	31.0		
β-ZEL	319.2	275.2 ^1^	21.0	13.3	11.9
		160.3	33.0		
α-ZEL	319.2	275.2 ^1^	21.0	14.6	11.1
		160.3	33.0		
ZAN	319.2	275.2 ^1^	23.0	14.9	93.1
		205.2	24.0		
β-ZAL	321.1	277.2 ^1^	23.0	12.7	29.3
		303.2	22.0		
α-ZAL	321.1	277.2 ^1^	23.0	14.3	28.8
		303.2	22.0		
ZEN-14GlcA	493.1	317.0 ^1^	31.0	8.8	40.3
		175.0	21.0		
β-ZEL-14GlcA	495.1	319.0 ^1^	22.0	7.4	40.5
		113.0	28.0		
α-ZEL-14GlcA	495.1	319.0 ^1^	22.0	8.5	35.0
		113.0	28.0		
C13	335.0	185.0 ^1^	27.0	15.2	93.3
		140.0	34.0		

^1^ Quantifying ion; ^2^ defined as the peak area of qualifier in percent of quantifier.
